# Rapid activity-dependent modulation of the intrinsic excitability through up-regulation of KCNQ/Kv7 channel function in neonatal spinal motoneurons

**DOI:** 10.1371/journal.pone.0193948

**Published:** 2018-03-26

**Authors:** Joseph Lombardo, Jianli Sun, Melissa A. Harrington

**Affiliations:** Department of Biology, Delaware State University, Dover, Delaware, United States of America; University of Texas Health Science Center, UNITED STATES

## Abstract

Activity-dependent changes in the properties of the motor system underlie the necessary adjustments in its responsiveness on the basis of the environmental and developmental demands of the organism. Although plastic changes in the properties of the spinal cord have historically been neglected because of the archaic belief that the spinal cord is constituted by a hardwired network that simply relays information to muscles, plenty of evidence has been accumulated showing that synapses impinging on spinal motoneurons undergo short- and long-term plasticity. In the brain, brief changes in the activity level of the network have been shown to be paralleled by changes in the intrinsic excitability of the neurons and are suggested to either reinforce or stabilize the changes at the synaptic level. However, rapid activity-dependent changes in the intrinsic properties of spinal motoneurons have never been reported. In this study, we show that in neonatal mice the intrinsic excitability of spinal motoneurons is depressed after relatively brief but sustained changes in the spinal cord network activity. Using electrophysiological techniques together with specific pharmacological blockers of KCNQ/Kv7 channels, we demonstrate their involvement in the reduction of the intrinsic excitability of spinal motoneurons. This action results from an increased M-current, the product of the activation of KCNQ/Kv7 channels, which leads to a hyperpolarization of the resting membrane potential and a decrease in the input resistance of spinal motoneurons. Computer simulations showed that specific up-regulations in KCNQ/Kv7 channels functions lead to a modulation of the intrinsic excitability of spinal motoneurons as observed experimentally. These results indicate that KCNQ/Kv7 channels play a fundamental role in the activity-dependent modulation of the excitability of spinal motoneurons.

## Introduction

The potential for long-term plastic changes in the properties of the spinal motor system has long been doubted because of the archaic belief that the spinal cord is constituted of hardwired networks that simply relay information to muscles [1]. However, frequency-dependent changes in synaptic efficacy were first demonstrated in synapses of the reflex pathway impinging on spinal motoneurons (MN) [2]. Although short-term modifications in the properties of the locomotor central pattern generator (CPG) synapses on MNs have been reported [3–5], long-term changes are less likely, in particular after development, due to the stereotyped nature of locomotion. However, activity-dependent modifications of the descending and the sensory-motor pathways impinging on MNs might instead be crucial for resetting the properties of the motor system appropriately, according to the environmental and developmental demands of the organism [1]. Maladaptive plasticity has also been observed in pathological conditions that affect spinal MNs [6–8], further underlining the importance of understanding these processes.

In the brain, brief changes in the activity level of the network, such as those necessary to trigger short and long-term synaptic plasticity, have been shown to be paralleled by changes in the neuronal intrinsic excitability, and are mainly suggested to reinforce the changes at the synaptic level. In contrast, chronic changes in the network activity are believed to induce homeostatic changes that contribute to stabilize the network activity [9, 10]. Several investigations have illustrated that the intrinsic excitability of MNs is modulated in the long term after physical training [11], operant conditioning [12], weight bearing [13], as well as after chronic changes in the spinal cord activity [14–16]. Rapid activity-dependent changes in the properties of synapses impinging on spinal MNs have also been established [1, 17]. Besides changes in the intrinsic electrical properties of spinal MNs in the crayfish and *Xenopus* MNs [18–20], however, modifications in the intrinsic properties of mammalian spinal MNs after brief changes in network activity have not been reported. Since the output of MNs depends on the interaction between the synaptic strength of the connections impinging on them and their intrinsic excitability, understanding the full impact of plastic changes in spinal MNs requires identifying the extent that their intrinsic excitability undergoes relatively rapidly-induced plastic changes.

To determine the extent to which spinal MNs can undergo plastic changes of their intrinsic properties, we have investigated the impact of a prolonged increase in the activity of the spinal network on lumbar MNs in neonatal mice. Using a combination of electrophysiological recordings, pharmacological treatments and computational modeling, we demonstrate that a relatively short but sustained increase in spinal network activity enhances the activity of KCNQ/Kv7 channels in spinal MNs leading to a reduction in their intrinsic excitability.

## Materials and methods

### Animals and slice preparation

All animal experiments were conducted according to a protocol approved by Delaware State University’s Institutional Animal Care and Use Committee, and were in accordance with NIH guidelines. Mouse pups at post-natal day three to eight (P3 –P8) were decapitated and the spinal cord was obtained as described previously [21]. Briefly, a filed needle of appropriate size was inserted into the caudal side of the spinal column and the spinal cord was squirted out with the aid of a syringe filled with ice-cold dissecting solution (concentrations in mM: 22 NaCl, 1.8 KCl, 1.2 NaH_2_PO_4_, 108 sucrose, 25 NaHCO_3_, 0.5 CaCl_2_, 3.2 MgCl_2_, 36 glucose) bubbled with 95% O_2_/5% CO_2_ (pH 7.4, 310–330 mOsm) until use. The spinal cord was then pinched in a groove of a 5% agar block which was then glued in the chamber of a Vibroslice NVSLM1 slicer from World Precision Instruments (Sarasota, FL), and 400-μm-thick slices were cut from the lumbar enlargement. The slices were then transferred into a custom made chamber kept at 34°C to promote their recovery and filled with artificial cerebro-spinal fluid (ACSF) containing (in mM) 117 NaCl, 36 glucose, 25 NaHCO_3_, 3.6 KCl, 1.2 NaH_2_PO_4_, 2.5 CaCl_2_, 1.2 MgCl_2_, continuously bubbled with 95% O_2_/5% CO_2_ (pH 7.4, 310–330 mOsm). After 30 min, the slices were brought at room temperature until used to slow down their decay.

### MEA recordings and high K^+^ treatment

Multi-electrode array (MEA) recordings were performed at room temperature with the Med64 system from Alpha Med Scientific (Osaka, Japan). The solution in the recording chamber was continuously bubbled with 95% O_2_/5% CO_2_. Recordings were made for a 3 min periods (in control ACSF, after 15 and 27 min in high K^+^, and after return to control ACSF). Spikes were detected with a 10 μV threshold and extracted with Mobius software from Alpha Med Scientific applying a 30% cluster similarity radius for their assignment to the same active unit [22]. Glucose was equimolar reduced when KCl was increased to 10 mM (i.e. high K^+^); however, its concentration remained an order of magnitude higher than values reported to induce changes in the activity of “glucose-sensitive” proteins [23–25].

### Whole-cell patch-clamp recordings

The recording chamber was continuously perfused at a rate of 2–3 ml/min with pre-heated ACSF solution at 34°C to reproduce the physiological temperature in mice. Large ventral horns cells with a soma axis larger than 20 μm were identified using an IR-1000 infrared sensitive camera from DAGE MTI (Michigan City, IN) coupled with a DIC-equipped BX51WI microscope from Olympus (Center Valley, PA). These cells were then patched in the whole-cell configuration. Current- and voltage-clamp recordings were acquired with a MultiClamp 700B amplifier at 10 kHz and filtered at 3 kHz with a Digidata 1440A digitalizer (both from Molecular Devices; Sunnyvale, CA). The patch pipettes were filled with an internal solution containing either (in mM) 140 KCl, 10 HEPES, 4 MgATP, 2 MgCl_2_, 0.5 EGTA, and 0.5 NaGTP for current-clamp recordings or (in mM) 140 KGluconate, 10 HEPES, 11 EGTA, 2 MgCl_2_, 2 CaCl_2_, and 4 NaATP, and 0.2 NaGTP for voltage-clamp recordings (pH 7.2–7.3 with KOH, 290–310 mOsm). The initial open-tip resistance of the patch pipettes was 2–5 MΩ. In current-clamp recordings, synaptic currents were suppressed by supplementing the ACSF with (in μM) 10 CNQX, 50 LD-AP-5, 10 bicuculline, 1 CPG 55845, and 10 strychnine to block AMPA, NMDA, GABA_A_, GABA_B_, and glycine receptors, respectively. In voltage-clamp recordings, CaCl_2_ was removed from the extracellular solution to eliminate calcium-dependent currents, and TTX (1 μM) was added to block voltage-gated sodium channels. Broad blockers of calcium channels were omitted because of their reported inhibitory effect on the M-current [26, 27]. All drugs, including XE991, were purchased from Tocris (Bristol, UK), except from strychnine which was purchased from Sigma-Aldrich (St. Louis, MO).

### Analysis

For the analysis of the network activity we included slices that showed spiking activity in control ACSF or in high K^+^ (all 19 slices tested met such condition). For patch clamp analysis, we retained recordings that, prior to the application of synaptic blockers, had a stable membrane potential equal or below– 50 mV, sustained action potential firing with high current injections, and the presence of a distinct afterdepolarization potential. These properties have been ascribed to spinal MNs and, considered together, are not present in spinal interneurons [28, 29]. Series resistance averaged ~ 13 MΩ and recordings with more than a 5 MΩ change were discarded. Neither series resistance nor liquid junction potentials were compensated for in current-clamp, whereas a 13.5 mV junction potential was subtracted in voltage-clamp. Input resistance was defined as the inverse of the slope conductance which was obtained at– 70 mV by linear fitting procedures of the current-voltage (*I*–*V*) curve in the sub-threshold range of membrane potentials (– 400 pA to + 100 pA) with 500-ms pulses (50-pA steps). Single action potentials (APs) were elicited from the common– 70 mV membrane potential by brief 25-ms square pulses (10-pA steps) and AP properties were analyzed at the minimal 25-ms step current injection necessary to trigger an AP. The voltage threshold was defined as the first peak in the third time derivative of the membrane potential [30], whereas the AP amplitude was measured from the peak of the overshoot to the peak of the fast after-hyperpolarization. The M-current was recorded with the standard deactivation protocol with P/-4 online leak subtraction, as defined in the pClamp software from Molecular Devices, and its isolation was confirmed by its blocker XE991 (20 μM). The peak amplitude of the current was defined as the difference of the activated current at the end of the pre-step and the average sustained current of the last 50 ms of each voltage step. Values are reported as mean ± s.e.m to highlight the separation between the means in the different conditions. *P* values were obtained with appropriate statistical tests as reported.

### Simulations

We used a computational model previously developed to account for the pharmacological modulations of KCNQ/Kv7 channels in neonatal spinal MNs (http://senselab.med.yale.edu, accession #:217882) [21]. The files are available for public download under the ModelDB section of the SenseLab database (http://senselab.med.yale.edu).

## Results

### High K^+^ increases the spinal network activity in a sustained and reversible fashion in acute neonatal mouse spinal cord slices

**Fig 1:**
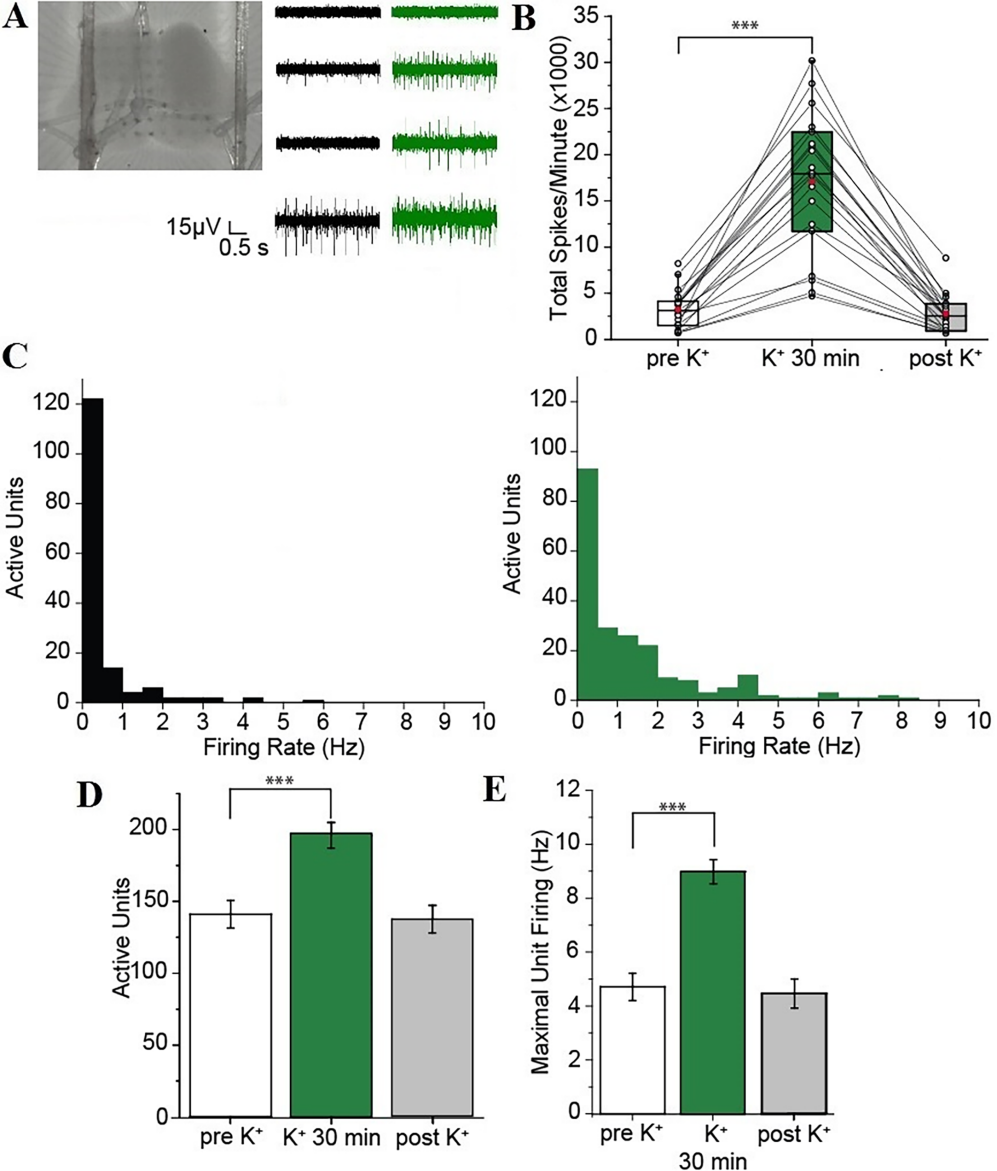
High K^+^ induces a sustained and reversible increase of spinal network activity. **(**A) Acute spinal cord slices from the lumbar enlargement of a neonatal mouse placed over the MEA (*left*) and a representative activity over four adjacent electrodes in low (*black*) and high K^+^ (*green*). (B) Box plot with the total number of spikes (in thousands) per minute in control ACSF (*white*), high K^+^ (*green*), and after returning to control ACSF (*gray*). Each line represents an individual slice. Red dots show the means under the three different conditions. (C) Histograms of the number of active units firing at different average frequencies in control ACSF (*black*) and high K^+^ (*green*). (D-E) Summary diagrams comparing the number of active units (D) and the maximal unit firing rate (E) in control ACSF (*white*), high K^+^ (*green*), and after returning to control ACSF (*gray*). Error bars, s.e.m. ***, *P* ≤ 0.001. *P* values were obtained with related-samples Friedman’s two-way ANOVA by ranks with Bonferroni correction for multiple corrections.

To elicit a sustained increase in the activity of spinal networks, we incubated acute slices obtained from the lumbar spinal cord of neonatal mice (postnatal days 3–8) in elevated K^+^ for ≥ 30 min (see METHODS). The activity of the lumbar spinal network under these conditions was recorded with multi-electrode arrays (MEA) (Fig 1).

In control ACSF, the spinal cord network was characterized by a variable level of activity with some electrodes showing no activity and others showing both low and high amplitude activity (Fig 1A). Compared to controls, the total number of spikes (across all electrodes) per minute was significantly increased in high K^+^ (3.24 ± 0.48 × 10^3^ spikes/minute versus 16.88 ± 1.86 × 10^3^ spikes/minute, *n* = 19; *P* < 0.001; Fig 1). The spinal network activity in the high K^+^ solution remained elevated and was not significantly reduced even after 30 min compared to controls (17.07 ± 1.74 × 10^3^ spikes/minute, *n* = 19; *P* = 1). After returning the spinal cord slices to the control ACSF, the spontaneous spinal network activity returned to the initial control level (2.78 ± 0.45 × 10^3^ spikes/minute, *n* = 19; *P* = 1; Fig 1B), demonstrating that the increase in the extracellular K^+^ concentration can be effective in sustainably but reversibly enhancing the spinal network activity for a prolonged period of time. Although an analysis of the regional-specific activity in the spinal cord was not attempted due to the potential tissue damage induced by the ensuing necessary reorientation of the slices over the MEAs, the effect of high K^+^ could be further analyzed with spike sorting and clustering at the single unit level represented by each, sorted cluster of spikes thanks to the low cell density present in acute slices [22, 31]. Compared to controls, the number of separated active units firing APs was significantly increased in high K^+^ (141.05 ± 9.62 versus 195.95 ± 8.87, *n* = 19; *P* < 0.001; Fig 1E). In addition, the maximal firing rate of each unit was also significantly increased (4.71 ± 0.51 Hz versus 9.05 ± 0.45 Hz, *n* = 19; *P* < 0.001; Fig 1E).

Taken together these results suggest that exposing neonatal mouse acute spinal cord slices to high K^+^ effectively and consistently enhances the network activity sustainably and in a reversible fashion both by recruiting silent neurons and increasing the firing rate of the already active ones.

### Prolonged enhancement of the spinal network activity reduces the intrinsic excitability of spinal motoneurons

**Fig 2:**
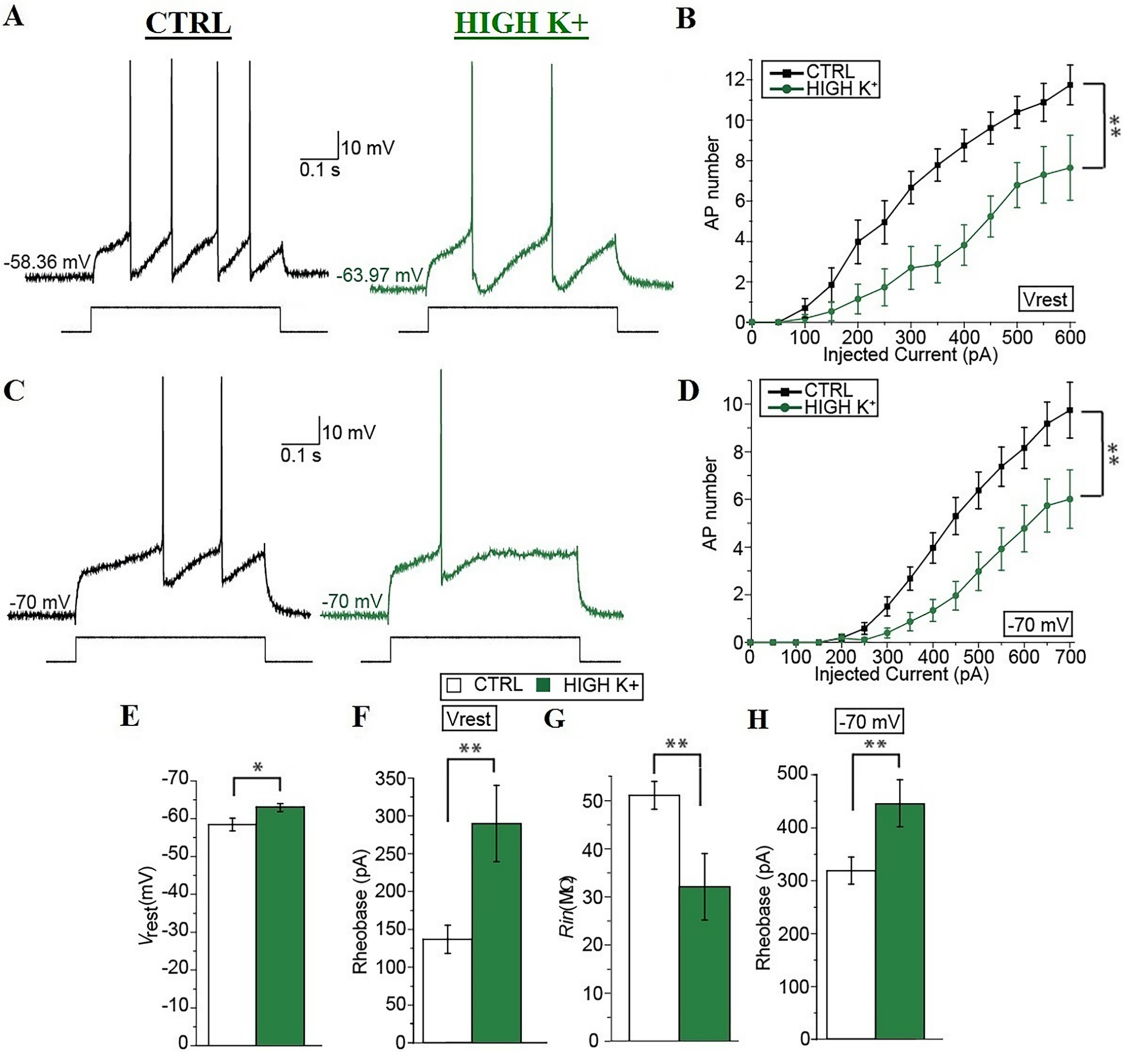
Prolonged enhancement of spinal network activity decreases the intrinsic excitability of spinal motoneurons. (A) Representative recordings obtained at the different resting membrane potential of the neurons in control (black) and after ≥ 30 min in high K^+^ (green) with a current pulse of the same intensity (i.e. 300 pA, shown below). (B) Input-output curves in the different conditions reporting the different number of APs fired after injecting 500-ms depolarizing current pulses of different levels (0–600 pA, 50-pA steps). (C) Representative recordings obtained at– 70 mV in control (black) and after ≥ 30 min in high K^+^ (green) with a current pulse of the same intensity (i.e. 300 pA, shown below). (D) Input-output curves in the different conditions reporting the different number of APs fired after injecting 500-ms depolarizing current pulses of different levels (0–700 pA, 50-pA steps) from a common membrane potential of– 70 mV obtained through appropriate DC injections. (E–H) Summary diagrams comparing the resting membrane potential (*V*_rest_; E), the rheobase of the neurons at their resting membrane potentials (F), the input resistance (*R*_in_; G), and the rheobase of the neurons at the common membrane potential of– 70 mV (F) in control (white) and after ≥ 30 min in high K^+^ (green). Error bars, s.e.m. *, *P* < 0.05; **, *P* < 0.01. *P* values were obtained with Mann-Whitney-Wilcoxon test, except from input-output (*I*–*O*) curves which were analyzed with repeated two-way ANOVA.

Increasing network activity has been shown to modify the intrinsic properties of principal neurons in the brain, however little information has been obtained about changes in the intrinsic properties of neurons in the spinal cord, especially in mammals [32]. We thus compared the intrinsic excitability of mouse spinal MNs in spinal cord slices after ≥ 30 min of their exposure to control solution or high K^+^ solution. Consistent with studies of other CNS principal neurons [33], the input-output (I-O) curve of neonatal mouse spinal MNs was significantly shifted towards higher input currents after ≥ 30 min in high K^+^ (between subjects: *P* < 0.01; Fig 2B).

**Fig 3:**
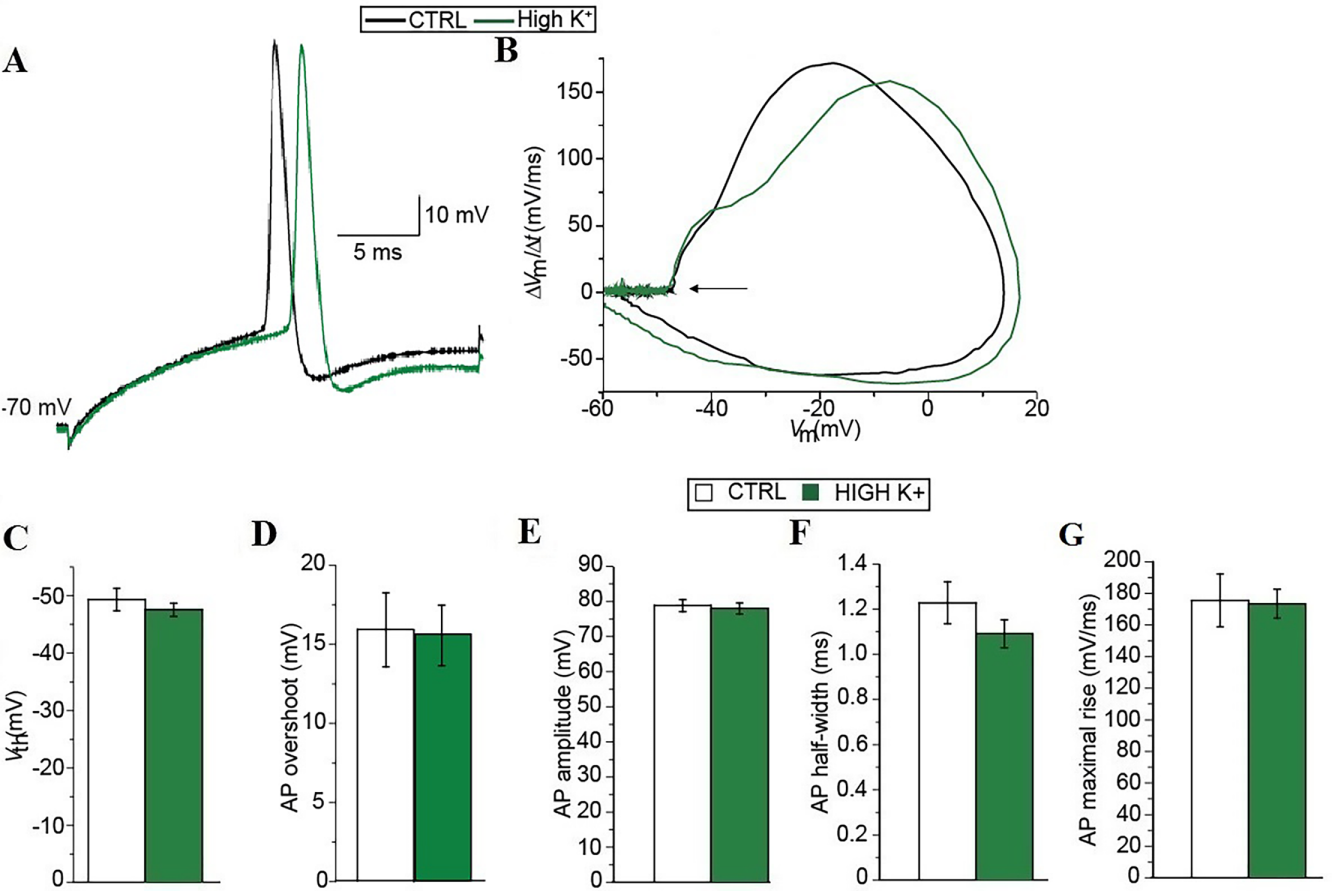
Single action potential properties are not modified by enhancements of spinal network activity. Single action potentials were evoked by brief 25-ms depolarizing current pulses of different levels (10-pA steps) from a common membrane potential of– 70 mV obtained through appropriate DC injections. (A) Representative recordings obtained at– 70 mV in control (black) and after ≥ 30 min in high K^+^ (green). (B) First time derivative of the membrane potential plotted against the membrane potential shows the membrane potential trajectory and the arrow highlights the potential at which the first time derivative deviates from 0 in control (black solid line) and after ≥ 30 min in high K^+^ (green solid line). (C–G) Summary diagrams comparing the voltage threshold (*V*_th_; C), the action potential overshoot (D), the action potential amplitude (E), the action potential maximal rate of rise (F), and the action potential half-width (G) in control (white) and after ≥ 30 min in high K^+^ (green). Statistical significance was evaluated with the Mann-Whitney-Wilcoxon test.

After ≥ 30 min in high K^+^, in particular, the resting membrane potential of spinal MNs was significantly hyperpolarized compared to controls (– 62.64 ± 1.09 mV versus– 58.05 ± 1.67 mV, *n* = 13 and *n* = 10, respectively; *P* = 0.032; Fig 2A and 2E). Also, the rheobase measured at the different resting membrane potential of the cells was significantly higher after high K^+^ incubation (289 ± 50 pA versus 136 ± 19 pA, *n* = 15 and *n* = 10, respectively; *P* = 0.002; Fig 2F). Since the higher rheobase after ≥ 30 min in high K^+^ could simply result from the more hyperpolarized resting membrane potential of the cells in this condition, we compared the intrinsic excitability of spinal MNs in the two conditions at a common potential of– 70 mV obtained by DC current injections through the patch pipette. Consistent with our results at the resting membrane potential, the number of APs produced by depolarizing steps was significantly decreased in neurons incubated ≥ 30 min in high K^+^ compared to neurons in control solution (between subjects: *P* < 0.01; Fig 2C and 2D). Also, the rheobase measured at a common potential of– 70 mV was significantly higher after ≥ 30 min in high K^+^ compared to controls (443 ± 44 pA versus 317 ± 26 pA, *n* = 16 and *n* = 10, respectively; *P* = 0.004; Fig 2H). Finally, the input resistance (*R*_in_) after ≥ 30 min in high K^+^ was significantly decreased compared to controls (31.83 ± 6.90 MΩ versus 50.83 ± 2.88 MΩ; *n* = 17 and *n* = 12, respectively; *P* = 0.003; Fig 2G). We also compared the properties of single APs triggered by minimal brief (25 ms) current injections necessary to trigger single AP after ≥ 30 min in control or in high K^+^ (Fig 3).

Single AP properties appeared to show quite some cell-to-cell variability (Fig 3A). In particular, the average AP threshold after ≥ 30 min in high K^+^ was slightly more depolarized compared to controls (– 47.41 ± 1.04 mV versus– 48.79 ± 1.70 mV; *n* = 13 and *n* = 12, respectively; Fig 3C); however, it did not result to be statistically significant (*P* = 0.349). This aspect was also highlighted in the phase plot analysis where traces differed in their overall shape, most likely because of the structural cell-to-cell variability [34], but tended to start to deviate from 0 at the same level (arrow in Fig 3B). Also other properties of single APs, such as their overshoot (Fig 3D), amplitude (Fig 3E), half-width (Fig 3F), and maximal rate of rise (Fig 3G) were not significantly different after ≥ 30 min in high K^+^ compared to controls. Taken together these results suggest that the reduction of the intrinsic excitability of neonatal mouse spinal MNs is due mainly to a hyperpolarization of their resting membrane potential and a decrease in their input resistance.

### Prolonged spinal network enhancement decreases the intrinsic excitability of spinal motoneurons through an up-regulation of their the M-current

**Fig 4:**
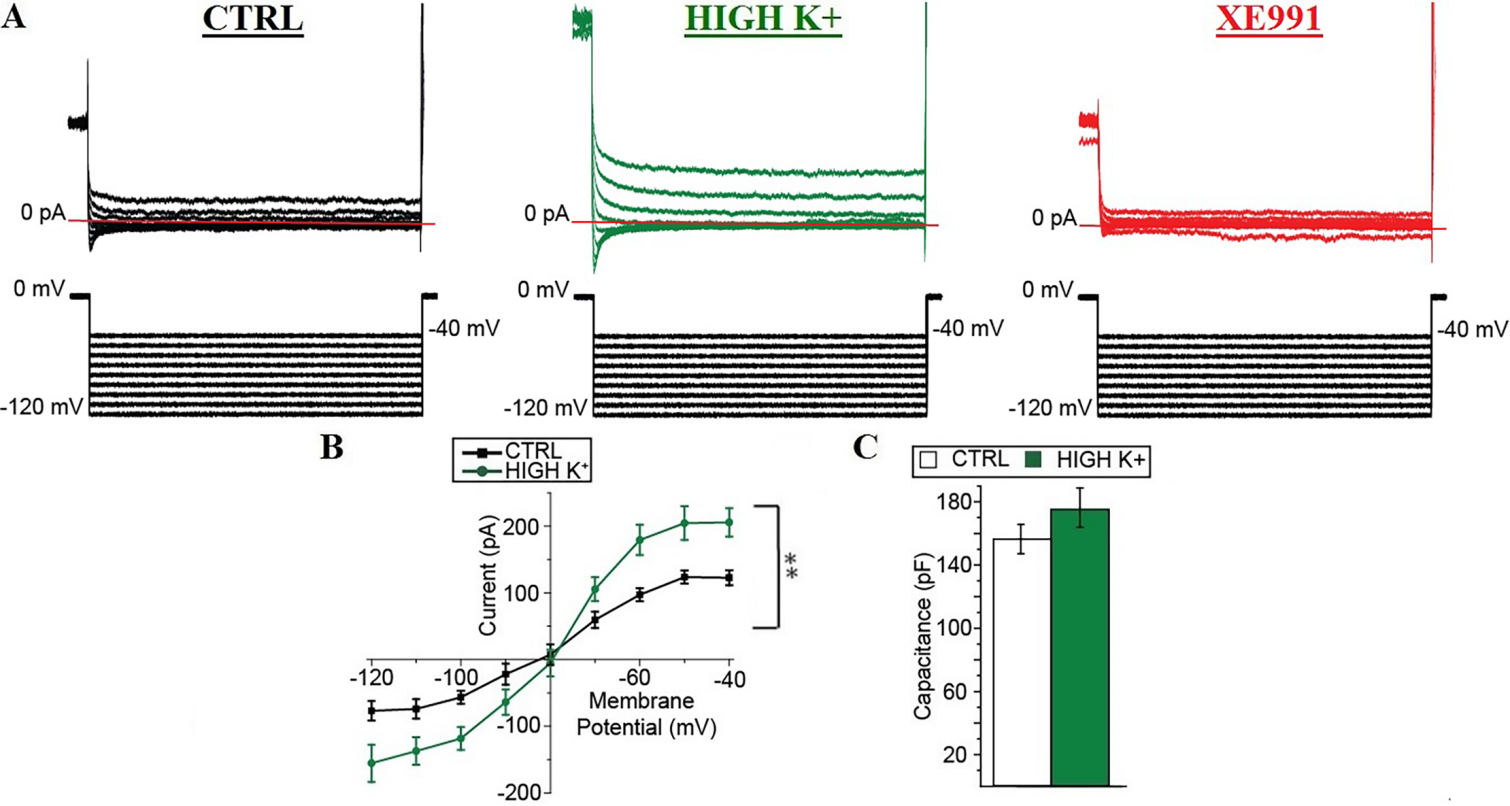
Prolonged enhancement of spinal network activity increases the M-current in spinal motoneurons. (A) Representative current recordings obtained in control (black) neurons and in neurons after ≥ 30 min in high K^+^ with (red) and without XE991, a specific blocker of KCNQ/Kv7 channels (green) at different membrane potentials (from– 40 mV to– 120 mV, 10-mV steps, as shown below) after a 8 s pre-pulse at 0 mV. In red is shown the zero current level. (B) Current-voltage (*I*–*V*) plot in the different conditions reporting the different level of the leak subtracted current at the different membrane potentials. (C) Diagram comparing the membrane capacitance of the neurons in control conditions (white) and after ≥ 30 min in high K^+^ (green). Error bars, s.e.m. **, *P* < 0.01. *P* value of the current-voltage (*I*–*V*) curves was analyzed with repeated two-way ANOVA.

Apart from the lack of significance in the change in the AP voltage threshold, the changes in the intrinsic excitability observed after ≥ 30 min in high K^+^ resembled the changes induced in MNs by the pharmacological enhancement of the function of KCNQ/Kv7 channels [21, 35]. In addition, the function of KCNQ/Kv7 channels has been shown to undergo activity-dependent modulation in several types of CNS neurons [36–39]. This hinted at the possibility that the reduction in the intrinsic excitability of spinal MNs could be the consequence of the activity-dependent enhancement of KCNQ/Kv7 channel function. In order to test whether the change in the properties of spinal MNs was the consequence of a modulation of KCNQ/Kv7 channels, we recorded the M-current, i.e. the current produced by the activition of KCNQ/Kv7 channels, in voltage-clamp in spinal MNs after ≥ 30 min in control conditions or in high K^+^ (Fig 4).

After ≥ 30 min in high K^+^, the current recorded in spinal MNs with the deactivation protocol appeared to be larger, both at the end of the pre-pulse step at 0 mV and at most membrane potentials after that the current fully relaxed (Fig 4A). Indeed, the slope of the current-voltage (I-V) curve of neonatal mouse spinal MNs was significantly increased compared to controls (between subjects: *P* < 0.01; Fig 4A and 4B). However, the membrane capacitance of spinal MNs did not appear to be significantly different after ≥ 30 min in high K^+^ compared to controls (175 ± 12 pF versus 156 ± 9 pF, *n* = 8 and *n* = 15, respectively; *P* = 0.23; Fig 4C), suggesting that the increased current at each voltage was not caused by a larger perisomatic area of the spinal MNs after the high K^+^ treatment. Taken together these results suggest that ~ 30 min activation of the spinal network significantly increases the M-current in neonatal mouse spinal MNs.

Consistent with our expectations, the M-current, i.e. the product of the activation of KCNQ/Kv7 channels, undergoes an activity-dependent increase in spinal MNs, suggesting that its modulation might underlie the reduction in the intrinsic excitability of these neurons. In other types of neurons, high K^+^ treatment has been shown to modulate the neuronal intrinsic excitability via several mechanisms [33, 40]. For example, an up-regulation in KCNQ/Kv7 channel function has recently been associated with an increased intrinsic excitability in brainstem auditory neurons when coupled with the modulation of other ion channels [41]. It would thus be possible that a modulation of other ion channels might coexist with the up-regulation of KCNQ/Kv7 channels and contribute to determining the intrinsic excitability of spinal MNs after high K^+^ treatment.

**Fig 5:**
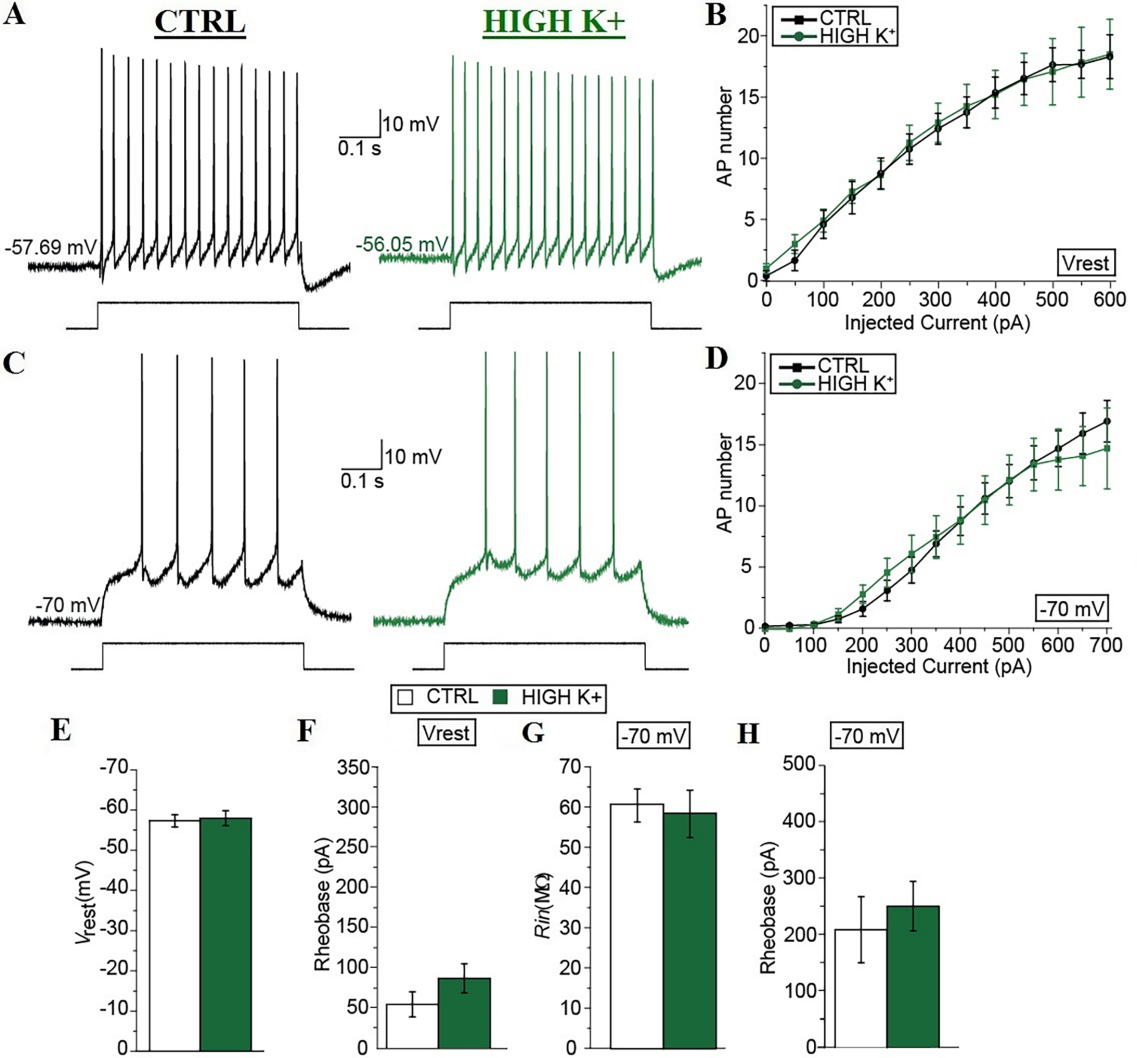
Blocking KCNQ/Kv7 channels eliminates the difference in the intrinsic excitability of spinal motoneurons induced by the prolonged enhancement of the spinal network activity. (A) Representative recordings obtained at the different resting membrane potential of the neurons in XE991 (black) or in the presence of XE991 after ≥ 30 min in high K^+^ (green) with a current pulse of the same intensity (i.e. 300 pA, shown below). (B) Input-output curves in the different conditions reporting the different number of APs fired by injecting 500-ms depolarizing current pulses of different levels (0–600 pA, 50-pA steps). (C) Representative recordings obtained at– 70 mV in XE991 (black) or in the presence of XE991 after ≥ 30 min in high K^+^ (green) with a current pulse of the same intensity (i.e. 300 pA, shown below). (D) Input-output curves in the different conditions reporting the different number of action potentials (AP) fired by injecting 500-ms depolarizing current pulses of different levels (0–700 pA, 50-pA steps) from a common membrane potential of– 70 mV obtained through appropriate DC injections. (E–H) Summary diagrams comparing the resting membrane potential (*V*_rest_; E), the rheobase of the neurons at the different resting membrane potentials (F), the input resistance (*R*_in_; G), and the rheobase of the neurons at the common membrane potential of– 70 mV (F) in XE991 (white) or in the presence of XE991 after ≥ 30 min in high K^+^ (green). Error bars, s.e.m. *P* values were obtained with Mann-Whitney-Wilcoxon test, except from input-output (*I*–*O*) curves which were analyzed with repeated two-way ANOVA.

In order to understand to what extent the reduction of the intrinsic excitability of spinal MNs is due to the modulation of KCNQ/Kv7 channels, we compared the intrinsic excitability of spinal MNs in the presence of XE991 (3 μM), a specific blocker of KCNQ/Kv7 channels [42], after ≥ 30 min in control conditions or in high K^+^ (Fig 5A).

In the presence of XE991, the intrinsic excitability of spinal MNs did not appear to be significantly different after ≥ 30 min in high K^+^ or control conditions (Fig 5A–5H). In particular, the input-output (I-O) curves of neonatal mouse spinal MNs in the presence of XE991 were almost indistinguishable after ≥ 30 min in high K^+^ compared to controls (Fig 5B), and the resting membrane potential of the spinal MNs not spontaneously active in the presence of XE991 was not significantly different after ≥ 30 min in high K^+^ compared to controls (– 57.89 ± 1.88 mV versus– 57.25 ± 1.53 mV, *n* = 8 and *n* = 9, respectively; Fig 5A and 5E). Also, the rheobase at rest under the two different conditions was not significantly different (86 ± 18 pA versus 54 ± 16 pA, *n* = 8 and *n* = 10, respectively; Fig 5F). A potential confounding effect is that in the presence of XE991, 4 out of 13 spinal MNs in control conditions and 1 out of 8 spinal MNs after ≥ 30 min in high K^+^ were spontaneously active. Therefore, we decided to compare the rheobase at a common, slightly hyperpolarized membrane potential where no cell was spontaneously active (Fig 5C, 5D, 5G and 5H). In the presence of XE991, the rheobase at the common potential of– 70 mV was not significantly different after ≥ 30 min in high K^+^ compared to controls (251 ± 44 pA versus 209± 59 pA, *n* = 13 and *n* = 10, respectively; Fig 5H). Finally, in the presence of XE991, the input resistance (*R*_in_) after ≥ 30 min in high K^+^ was also not significantly different compared to controls (58.56 ± 5.88 MΩ versus 60.80 ± 4.10 MΩ; *n* = 9 and *n* = 13, respectively; Fig 5G). Taken together these results suggest that the prolonged activation of the spinal network induces a reduction of the intrinsic excitability of neonatal mouse spinal MNs mainly through a modulation of KCNQ/Kv7 channels function, and that other modulations, if present, play a limited role.

### The up-regulation of KCNQ/Kv7 channels function in a computational model reproduces the effects of prolonged spinal network activation on the neonatal spinal MNs

In our patch clamp experiments, ~30 min activation of spinal networks induced a hyperpolarization of the resting membrane potential, an increase in the rheobase and a decrease in the input resistance of neonatal spinal MNs. We and others [21, 35] have shown that retigabine, a selective enhancer of the activity of KCNQ/Kv7 channels [43], induces a depolarization of the AP voltage threshold in MNs. We thus expected that a prolonged activation of the spinal network causing increased M-current and KCNQ/Kv7 channel activity would have produced a significant depolarization of the AP voltage threshold [21, 35]. However, the effect of increased network activity on the AP voltage threshold, while in the same direction as the effect of retigatibine, did not reach significance.

**Fig 6:**
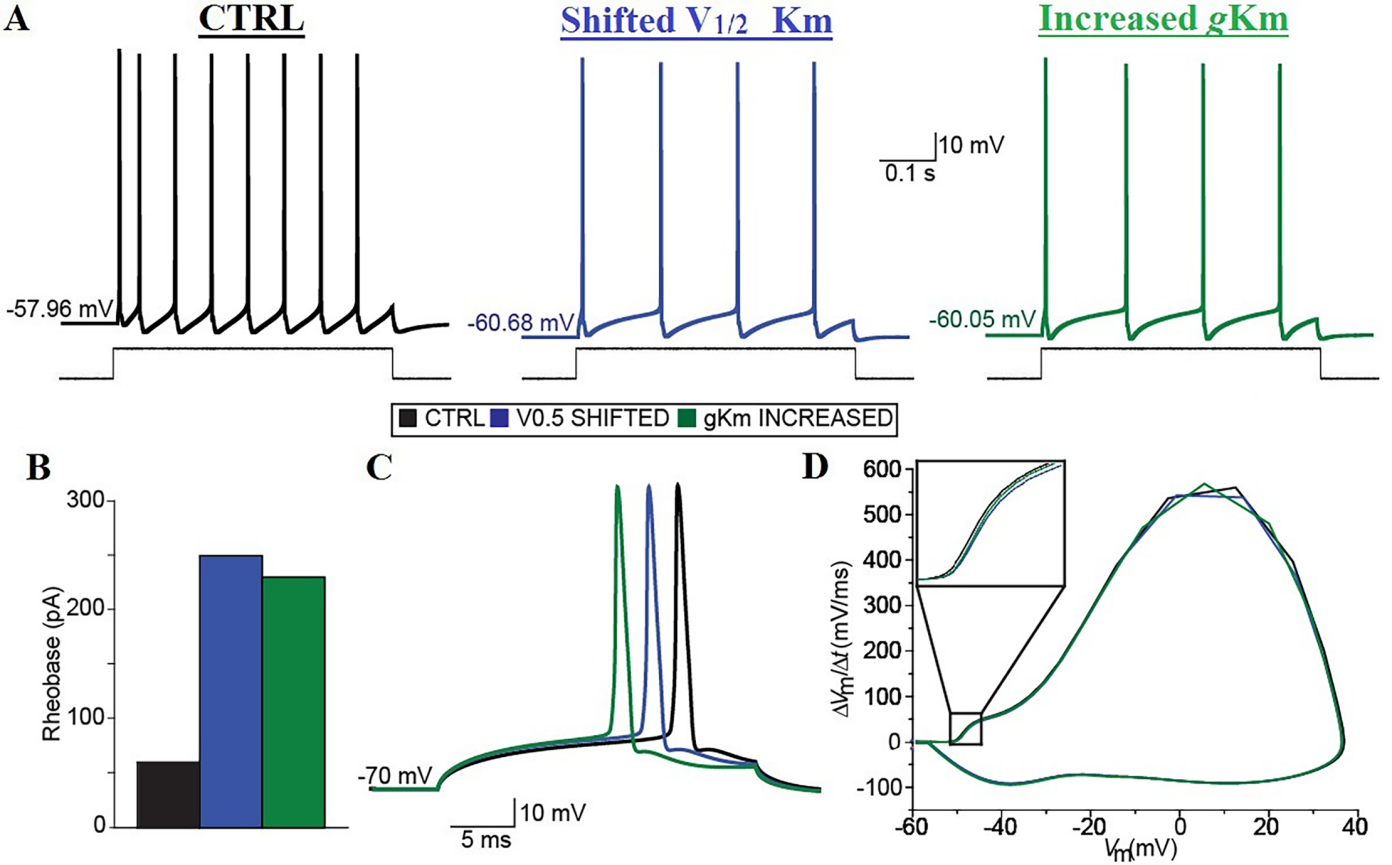
Up-regulations of KCNQ/Kv7 channels functions lead to changes in the intrinsic excitability of a computer model of spinal MNs similar to the reduction observed experimentally after high K^+^ treatment. (A) Action potential firing elicited in the model by simulating a 500-ms depolarizing current pulse injection of 300 pA in control (black), after shifting the model KCNQ/Kv7 channels half-activation voltage of– 7.25 mV (blue), and after increasing the conductance density of the model KCNQ/Kv7 channels 6 folds (green). (B) Rheobase of the model in control conditions (black), after shifting the model KCNQ/Kv7 channels half-activation voltage of– 7.25 mV (blue), and after increasing the conductance density of the model KCNQ/Kv7 channels 6 folds (green). (C) Single APs elicited in the model by simulating brief 25-ms depolarizing current pulses of different levels (10-pA steps) from a common membrane potential of– 70 mV in control conditions (black), after shifting the model KCNQ/Kv7 channels half-activation voltage of– 7.25 mV (blue), and after increasing the conductance density of the model KCNQ/Kv7 channels 6 folds (green). (D) First time derivative of the model membrane potential plotted against the membrane potential shows the membrane potential trajectory and highlights the membrane potential at which the first time derivative deviates from 0.

To explore how an up-regulation of KCNQ/Kv7 channel function might impact the intrinsic excitability of spinal MNs, we employed a computational model that we previously used to model pharmacological modulations of KCNQ/Kv7 channels in neonatal mouse spinal MNs [21]. To model the effect of ~30 min activation of the spinal network, we shifted the half activation voltage of the model KCNQ/Kv7 channels or increased their conductance density by the appropriate amount needed to obtain a change in AP firing as we observed experimentally after high K^+^ treatment at rest i.e. ~50% reduction with a 300 pA current injection (Fig 2B) and a rheobase of ~250 pA. The appropriate reduction in action potential firing and rheobase could be obtained with a ~7.25 mV shift of the model KCNQ/Kv7 channels activation voltage or with a 6 fold increase in their conductance density (Fig 6A and 6B).

To substantiate our computational results further, we decided to take advantage of the small change in the AP voltage threshold (i.e.< 2 mV) induced by ~30 min activation of the spinal network in our experimental conditions. We thus simulated the minimal, brief (25 ms) current injections necessary to trigger single AP in the model in the two different conditions, and obtained the phase plot of the single AP in each of the conditions (Fig 6C and 6D). In line with our experimental evidence, when the properties of the model KCNQ/Kv7 channels were modified in the same fashion that allowed us to obtain the appropriate AP firing and rheobase, the phase plots suggested a similar change in the AP voltage threshold to that induced by retigabine (Fig 6D). Although our computational model suggests that either a shift in the activation voltage of KCNQ/Kv7 channels or an increase in their density could lead to a change in the intrinsic excitability of spinal MNs similar to the one we observed experimentally after the prolonged activation of the spinal network, a ~7.25 mV shift in the activation voltage of KCNQ/Kv7 channels appear to be more plausible. Indeed, activity-dependent changes in the properties of the M-current have been previously suggested to shift its activation voltage [39].

## Discussion

In this study, we described a novel, relatively rapidly-induced, activity-dependent mechanism that decreases the intrinsic excitability of spinal MNs following prolonged enhancement of spinal network activity. As evidenced by the hyperpolarized resting membrane potential, increased rheobase, and decreased input resistance, the responsiveness of spinal MNs was lowered after the increased activity of the spinal network. These changes in the intrinsic excitability observed after the high K^+^ treatment were abolished in the presence of XE991, a specific blocker of KCNQ/Kv7 channels, while the prolonged enhancement of network activity induced an increase in the M-current, that is produced by KCNQ/Kv7 channels. Finally, computer simulations in which the properties of KCNQ/Kv7 channels were modified to reproduce the changes in the intrinsic excitability of spinal MNs that we observe after high K^+^ treatment support the view that up-regulation of the function of these channels decreases intrinsic excitability with small changes in the AP voltage threshold, just as we observed experimentally.

### Increasing the network activity in the neonatal spinal cord

In the spinal cord, raising the extracellular K^+^ concentration has been shown to induce locomotor-like patterns of activity both in the whole spinal cord preparation [44] and in organotypic slices [45]. However, locomotor-like activity has not been obtained in acute spinal cord slices, most likely because intersegmental connections are necessary to induce the required alternating flexor/extensor activity [46]. In line with such observations, we did not observe alternating activity in our MEA recordings, however in the slices, the ventral horns show considerable spontaneous activity, and raising extracellular K^+^ results in sustained enhancement of the activity.

During both the pre- and post-natal period, spontaneous activity has been shown to play a crucial role in the development of the nervous system including the spinal cord [47]. In prenatal development, for example, blockade of spontaneous activity with blockers of glutamate receptors or sodium channels prevents the maturation of the normal electrical phenotype of spinal MNs [48]. The spontaneous spinal cord activity is thus likely to interact with both the descending inputs from the brain and the proprioceptive inputs from the muscles to shape its mature connectivity structure [49, 50]. Interestingly, NMDA receptors are expressed at considerable levels during the first three postnatal weeks throughout most of the spinal cord [51], and in spinal MNs, in particular, NMDA receptors have been shown to mediate a maturational signal coming from the proprioceptive system [52]. Calcium influx through NMDA receptors could thus be a good candidate to mediate changes in the properties of the M-current during the neonatal period.

### Homeostatic activity-dependent changes in spinal motoneurons

Raising extracellular K^+^ can induce plastic changes both in cultured neurons [40] and *ex vivo* [32, 33]. In our case, a long-term, i.e. lasting at least the ~20–40 min of our recordings, reduction in the excitability of MNs followed increased spinal network activity induced by high K^+^. In the lamprey, mature spinal MNs have been shown to decrease their intrinsic excitability in response to increased network activity [32]. In that case, however, the changes in the intrinsic excitability of MNs were induced after the network activity had been altered for days, while we observe changes after approximately 30 minutes of elevated activity. Changes with a similar time course to ours have been previously obtained in the lamprey, though after lesions, making it difficult to interpret their relevance for the intact system [53]. In the hippocampus, however, intrinsic excitability is known to be changed following both prolonged and brief changes in the network activity [9, 33, 54]. In hippocampal slices, for example, brief exposure to high K^+^ induces a reduction of the intrinsic excitability of CA1 hippocampal neurons [33]. Similar to what was observed in hippocampal slices, we have found that prolonged exposure to high K^+^ induces a reduction of the intrinsic excitability of spinal MNs. Depending on the time frame of induction, changes in the intrinsic excitability of brain neurons have been suggested to reinforce the changes occurring at the synaptic level or to homeostatically promote network stability [10]. Changes in intrinsic excitability are known to co-occur with changes at the synaptic level, and these changes have been implicated in learning and memory [10, 55]. Different forms of synaptic plasticity have also been shown in the spinal cord, especially in synapses impinging on spinal MNs [1, 17, 56]. At 10 Hz, for example, synapses impinging on spinal MNs have been shown to undergo depression [1, 17]. It is thus possible that the decrease in the intrinsic excitability of spinal MNs might parallel a depression induced at the synaptic level. Homeostatic changes in the intrinsic excitability of CNS neurons have also been demonstrated after brief changes in the network activity [33, 54], and these changes have been shown to be bi-directional [32, 33, 54], raising the possibility that reducing the spinal network activity may similarly enhance the intrinsic excitability of spinal MNs.

### Location-dependent modulation of KCNQ/Kv7 channels

In CNS neurons, KCNQ/Kv7 channels are mainly located in the somatic and axonal compartments [57], and axonal KCNQ/Kv7 channels have been suggested to control the AP voltage threshold [21, 35, 58, 59]. In hippocampal neuronal cultures, axonal KCNQ/Kv7 channels, both at the axon initial segment (AIS) and in the nodes of Ranvier, have recently been shown to be very stable, even after modulation of the network activity [60, 61]. In our experiments, the small effect on the AP voltage threshold of MNs might suggest that the modulation of KCNQ/Kv7 channels involves mainly the soma. In certain CNS neuronal types, somatic KCNQ/Kv7 channels play a crucial role in modulating the intrinsic excitability [62]. The role of somatic KCNQ/Kv7 channels in principal cells, such as spinal MNs, is not clear but it is possible that they might have a preferred impact on APs back-propagating from the AIS. Selective modulation of back-propagating APs at the AIS has already been reported in layer V pyramidal neurons from the prefrontal cortex [63]. In addition, it has been shown that blocking KCNQ/Kv7 channels in layer V pyramidal neurons has opposite effects on antidromic and orthodromic APs [26]. Enhancement of the function of somatic KCNQ/Kv7 channels would be particularly attractive in light of a homeostatic function. The high K^+^ treatment in slices is likely to cause a massive increase in the activity of synapses impinging on the dendrites of spinal MNs which together with repetitive back-propagating APs might lead to an excessive potentiation or depression of synaptic efficacy. A reduction in the intrinsic excitability of the soma could protect neurons from excessive alterations in synaptic efficacy.

In conclusion, we have shown that in mouse neonates spinal MNs can undergo relatively rapid activity-dependent changes of their intrinsic excitability. In particular, we have shown that after a prolonged increase in the activity of the spinal network the M-current is up-regulated, and we have also implicated the increased activity of KCNQ/Kv7 channels in the down-regulation of the excitability of spinal MNs. This mechanism may primarily serve a protective function during the early post-natal period when high spontaneous activity in the developing motor system can result from the presence of over-abundant synapses. However, similar changes may occur after motor system development is complete, perhaps as a contributor to the mechanisms of fatigue, in which increased activating input is needed to maintain motor output and performance [64]. Future studies will be aimed at characterizing this activity-dependent modulation in mature spinal MNs and its potential contribution both to fatigue and to protecting spinal MNs from pathological hyperexcitability.

## Supporting information

S1 Table Data used to generate figures. Data tables are provided for all figure panels except for those with sample traces of raw data. In the supporting information Excel spreadsheet each figure panel is represented as a separate sheet.
(XLSX)
